# The deubiquitinating enzyme USP7 regulates the transcription factor Nrf1 by modulating its stability in response to toxic metal exposure

**DOI:** 10.1016/j.jbc.2021.100732

**Published:** 2021-04-30

**Authors:** John J.W. Han, Daniel V. Ho, Hyun M. Kim, Jun Y. Lee, Yerin S. Jeon, Jefferson Y. Chan

**Affiliations:** Department of Laboratory Medicine and Pathology, University of California, Irvine, D440 Medical Sciences, Irvine, California, USA

**Keywords:** cellular stress, transcription factor, protein stability, deubiquitinase, toxic metals, MDM2, mouse double minute 2 homolog, Nrf1, nuclear factor E2-related factor 1, O-GlcNAc, O-linked β-N-acetylglucosamine, USP7, ubiquitin specific protease 7

## Abstract

The nuclear factor E2-related factor 1 (Nrf1) transcription factor performs a critical role in regulating cellular homeostasis as part of the cellular stress response and drives the expression of antioxidants and detoxification enzymes among many other functions. Ubiquitination plays an important role in controlling the abundance and thus nuclear accumulation of Nrf1 proteins, but the regulatory enzymes that act on Nrf1 are not fully defined. Here, we identified ubiquitin specific protease 7 (USP7), a deubiquitinating enzyme, as a novel regulator of Nrf1 activity. We found that USP7 interacts with Nrf1a and TCF11—the two long protein isoforms of Nrf1. Expression of wildtype USP7, but not its catalytically defective mutant, resulted in decreased ubiquitination of TCF11 and Nrf1a, leading to their increased stability and increased transactivation of reporter gene expression by TCF11 and Nrf1a. In contrast, knockdown or pharmacologic inhibition of USP7 dramatically increased ubiquitination of TCF11 and Nrf1a and reduction of their steady state levels. Loss of USP7 function attenuated the induction of Nrf1 protein expression in response to treatment with arsenic and other toxic metals, and inhibition of USP7 activity significantly sensitized cells to arsenic treatment. Collectively, these findings suggest that USP7 may act to modulate abundance of Nrf1 protein to induce gene expression in response to toxic metal exposure.

Nuclear factor erythroid 2 (NF-E2)-related factor-1 (Nrf1), also known as NFE2L1, is a transcription factor essential for the maintenance of cellular homeostasis. Genetic ablation of the Nrf1 gene in mice leads to embryonic lethality, and tissue-specific inactivation of Nrf1 using mice bearing the Cre/LoxP conditional *nrf1* null allele indicates that Nrf1 protects against neurodegeneration and development of steatohepatitis (1, 2, 3, 4). Nrf1 proteins form heterodimers with small MAF proteins and bind to *cis*-active sequences known as the antioxidant response element (5). Nrf1 has been shown to drive the expression of antioxidants and phase II detoxification enzymes, and it also plays an important role in controlling proteasome abundance by regulating coordinate transcription of proteasome genes under basal and activated conditions (3, 6, 7, 8, 9, 10). Aside from its role in the cellular stress response, Nrf1 also regulates osteoblast and odontoblast differentiation and hepatic lipid metabolism, and loss of Nrf1 function leads to genetic instability and promotes tumorigenesis (4, 11, 12, 13, 14).

The Nrf1 gene encodes several protein isoforms. Long isoforms of Nrf1 are made up of TCF11 and Nrf1a (also been designated previously as Nrf1). TCF11 consisting of 772 amino acids is the longest protein, whereas Nrf1a, composed of 742 amino acids, is a spliceoform missing residues 242 to 271 of TCF11 (15). Both TCF11 and Nrf1a share the same N-terminal domain that targets them to the endoplasmic reticulum where they are N-glycosylated. To gain entry into the nucleus, they must undergo retrograde translocation into the cytoplasm, where they are deglycosylated by NGLY1 (N-glycanase 1) and cleaved by DDI2 (DNA damage inducible 1 homolog 2). Normally, little Nrf1a and TCF11 accumulate because of proteasome-mediated degradation. It is thought that proteasome inhibition allows them to escape ubiquitination and degradation leading to their accumulation and import into the nucleus to mediate gene expression (16, 17, 18, 19, 20). Shorter protein isoforms of Nrf1, such as LCRF1 and Nrf1b, have also been described (21, 22, 23, 24). These smaller proteins lack the ER membrane targeting domain present in Nrf1a and TCF11. Although they are capable of mediating gene activation, their physiologic functions are currently unknown.

The ubiquitin-mediated protein degradation pathway plays an important role in controlling the abundance of Nrf1 proteins (18, 25, 26). Several E3 ubiquitin ligases have been identified to regulate ubiquitination of TCF11 and Nrf1a. Deubiquitinating enzymes work in opposition to protein ubiquitination by catalyzing the deconjugation of ubiquitin molecules from proteins, and they are an important regulatory mechanism controlling the function and abundance of proteins in a broad range of cellular processes (27). Currently, there are more than a hundred deubiquitinating enzymes that have been identified. Ubiquitin-specific protease 7 (USP7), also known as herpesvirus associated USP, belongs to the largest ubiquitin-specific protease subfamily (28, 29, 30). Although USP7 was originally identified through its interaction with ICP0 (infected cell polypeptide 0), a herpes viral protein, USP7 has been shown to play a key role in regulating the stability of tumor suppressor p53 and its E3 ubiquitin ligase, mouse double minute 2 homolog (MDM2), as well as proteins involved in multiple cellular pathways including various aspects of the cell cycle, immune response, cell survival, development, tumorigenesis, and viral infection (31, 32, 33, 34, 35, 36).

In this study, we found USP7 to copurify with Nrf1a, one of the long isoforms, in a screen to identify Nrf1-interacting proteins. We show here that USP7 counteracts ubiquitination and promotes stabilization of Nrf1a and TCF11. USP7 enhances TCF11- and Nrf1a-mediated transcriptional activation. Expression of TCF11 and Nrf1a by toxic heavy metal exposure is blunted in USP7 knockout cells, and inhibition of USP7 function blocked Nrf1a-mediated protection against arsenic-induced cytotoxicity. This study demonstrates that USP7 is a determinant of Nrf1 expression.

## Results

### TCF11 and Nrf1a interact with USP7

**Figure 1:**
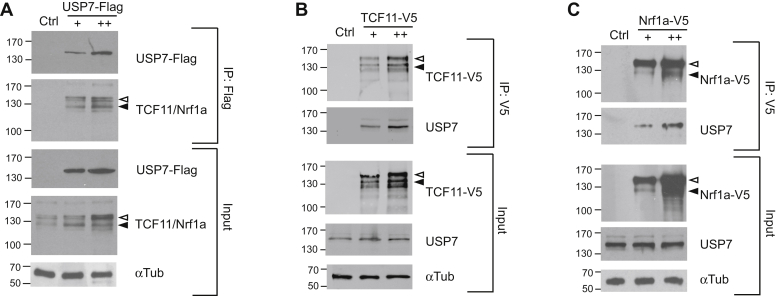
**Nrf1a and TCF11 interacts with USP7.** The interaction between Nrf1a and USP7 was tested by coimmunoprecipitation. *A*, HEK293 cells were transfected with increasing amount of Flag-tagged USP7 (0, 2, 4 μg). Cell lysates were prepared and immunoprecipitated using the anti-Flag Ab followed by immunoblotting of endogenous TCF11 and Nrf1a using anti-TCF11, which detects both long isoforms of Nrf1—TCF11 and Nrf1a. As input control, lysates were probed with anti-Flag, anti-TCF11, and anti-alpha tubulin antibodies. The membrane form (*open arrowhead*) of TCF11 and Nrf1a are seen as multiple bands migrating above 130 kDa, and membrane-free forms (*filled arrowhead*) are seen as bands migrating below 130 kDa. *B*, HEK293 cells were transfected with increasing amounts of V5-tagged TCF11 (0, 2, 4 μg), and cell lysates were prepared and immunoprecipitated using the anti-V5 mAb followed by immunoblotting using anti-USP7 Ab. As input control, cell lysates were immunoblotted with anti-V5 mAb, anti-USP7 Ab, and anti-alpha tubulin Ab. *C*, HEK293 cells were transfected with increasing amounts of V5-tagged Nrf1a (0, 2, 4 μg), and cell lysates were prepared and immunoprecipitated using the anti-V5 mAb followed by immunoblotting using anti-USP7 Ab. As input control, cell lysates were immunoblotted with anti-V5, anti-USP7, and anti-alpha tubulin antibodies.

In an effort to elucidate molecular mechanisms involved in regulating Nrf1 expression, we previously interrogated the Nrf1 interactome in cells by affinity purification coupled to mass spectrometry (37). USP7 was among the different proteins identified that copurified with streptavidin-tagged Nrf1a. To verify interaction with USP7, coimmunoprecipitation assays were done. We first examined whether endogenous Nrf1 proteins interact with USP7. HEK293 cells were transfected with increasing amounts of Flag-tagged USP7 expression plasmid, and lysates were immunoprecipitated with anti-Flag antibody followed by immunoblotting with anti-TCF11 antibody that detects both TCF11 and Nrf1a, which are the long isoforms of Nrf1. As shown in Figure 1*A*, Western blotting of immunoprecipitates prepared from USP7-Flag-transfected cells showed prominent bands just above and below the 130-kDa marker, which are the expected migration of the ER membrane-associated (∼120 kDa) and membrane-free (∼110 kDa) forms of TCF11 and Nrf1a (17, 18, 37). To examine whether endogenous USP7 interacts with the two different long isoforms of Nrf1, coimmunoprecipitations were performed on cells expressing V5-tagged TCF11 or V5-tagged Nrf1a. HEK293T cells were transfected with two different concentrations of TCF11-V5 or Nrf1a-V5 expression plasmid, and cell lysates were immunoprecipitated with anti-V5 antibody followed by immunoblotting with anti-USP7 antibody. As shown in Figure 1, *B* and *C*, endogenous USP7 was coimmunoprecipitated by TCF11-V5 and Nrf1a-V5 in a dose-dependent manner. Together, these results indicate that USP7 interacts with both long isoforms of Nrf1.

### TCF11 and Nrf1a are stabilized by USP7

**Figure 2:**
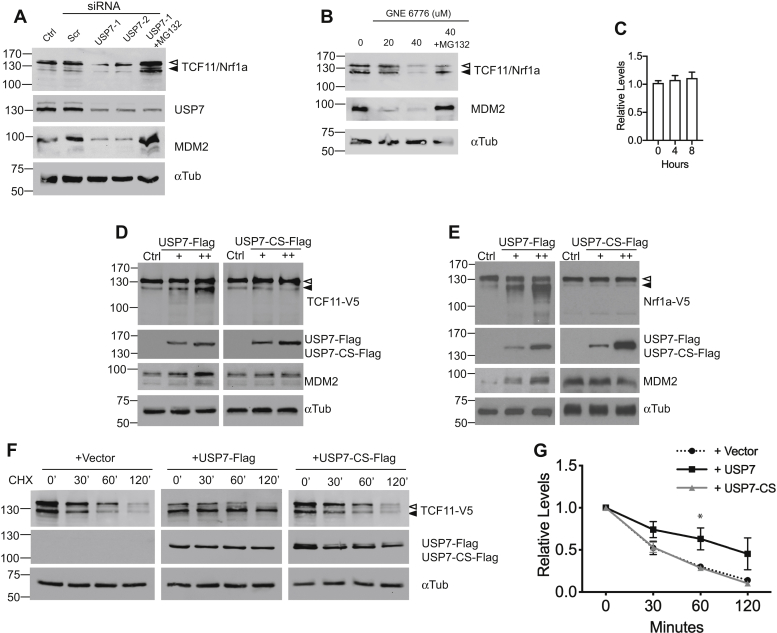
**TCF11 and Nrf1a are stabilized by USP7.***A*, HEK293 cells were transfected with 20 nM of two different siRNAs targeting USP7 (#1 or #2) or a negative control siRNA (Scr). One of the USP7-silenced sample was treated with 10 μM of MG132. Cell lysates were analyzed by immunoblotting against endogenous TCF11/Nrf1a and USP7. Lysates were also Western blotted with anti-MDM2 antibody as a control for USP7 activity and anti-alpha tubulin for loading. Unprocessed and processed forms of TCF11/Nrf1a are indicated by *open* or *filled arrowheads*, respectively. *B*, HEK293 cells were incubated overnight with 0, 20, or 40 μM of GNE-6776 and lysates prepared for Western blotting for endogenous TCF11/Nrf1a with anti-TCF11 antibody. Lysates were also probed with anti-MDM2 antibody as a control for USP7 activity and anti-alpha tubulin for loading control. *C*, HEK293 cells were cultured with 40 μM GNE-6776 for 0, 4, and 8 h and analyzed by RT-qPCR. Bar graph depicts relative expression of Nrf1 transcript normalized to untreated control (0 h), n = 3. HEK293 cells were transfected with 1 μg TCF11-V5 (*D*) or Nrf1a-V5 (*E*) along with increasing amounts of USP7-Flag (0.5, 1 μg) or USP7-CS-Flag (0.5, 1 μg). After 24 h, cell lysates were prepared for Western blotting with anti-V5 antibody. As input control, lysates were Western blotted with anti-Flag and anti-alpha tubulin antibodies. Cell lysates were also immunoblotted with anti-MDM2 antibody as a control for USP7 activity. *F*, HEK293 cells were transfected with 1 μg TCF11-V5 along with 1 μg vector control, USP7-Flag, or USP7-CS-Flag. After 24 h, cells were treated with cycloheximide (50 μg/ml), and lysates were prepared at the indicated time points and Western blotted with anti-V5 and anti-Flag antibodies. Loading of the lanes was determined by immunoblotting against alpha-tubulin. *G*, graph shows quantitation of cycloheximide chase assay monitoring Nrf1a-V5 stability. Experiments were performed on HEK293 cell lysates transfected with 1 μg Nrf1a-V5 along with 1 μg vector control, USP7-Flag, or USP7-CS-Flag followed by cycloheximide chase as described above. Samples were collected and subjected to Western blotting and densitometric quantification of Nrf1a-V5 relative to alpha-tubulin at each time point; time 0 was set to 100%. Statistical analysis was performed using Student’s *t* test. Each point represents the mean ± SEM of remaining protein for three independent experiments, ∗*p* value <0.05.

It is interesting to note that immunoblot analysis of cell lysates from cells transfected with USP7-Flag before immunoprecipitation showed increased steady-state levels of endogenous TCF11/Nrf1a in a dose-dependent manner (Fig. 1*A*, input control panel). This finding suggests that USP7 upregulates Nrf1 expression. To confirm this observation, we wish to determine if lowering endogenous USP7 level by RNA interference (siRNA)-mediated depletion of USP7 would reduce TCF11/Nrf1a expression. Two different siRNAs were used to silence USP7, and as shown in Figure 2*A*, knockdown of USP7 diminished endogenous TCF11/Nrf1a protein levels. Treatment with the proteasome inhibitor MG132 mitigated the effect of USP7 knockdown on TCF11/Nrf1a levels, suggesting that the effects of USP7 on TCF11/Nrf1a were dependent on the proteasome. As expected, MDM2, which is a known USP7 substrate, was decreased by RNAi-mediated knockdown of USP7 and this effect was also reversed by MG132. To corroborate the siRNA findings, the effects of GNE-6776, a small molecule compound known to selectively inhibit catalytic activity of USP7 (38), was also examined. Endogenous TCF11/Nrf1a levels, and MDM2 as control, were reduced by GNE-6776 and blocked by MG132 treatment (Fig. 2*B*). RT-qPCR shows that Nrf1 mRNA levels were not altered by GNE-6776 (Fig. 2*C*), indicating that the effects of USP7 on Nrf1 expression is at the protein level. To verify independently the effects of USP7 on TCF11 and Nrf1a, HEK293 cells were transfected with USP7-Flag along with TCF11-V5 or Nrf1a-V5 and their expression analyzed by Western blotting. Steady-state levels of bands corresponding to membrane-free (lower band) V5-tagged TCF11 and Nrf1a were significantly increased by overexpression of USP7-Flag in a dose-dependent manner (Fig. 2, *D* and *E*, left panels). As expected, transfection of USP7-Flag increased expression of endogenous MDM2. In contrast, steady-state levels of V5-tagged-TCF11 and Nrf1a, as well as endogenous MDM2, were unaffected by transfection of the catalytically inactive USP7-CS-Flag (Fig. 2, *D* and *E*, right panels). To measure the effects of USP7 on turnover of Nrf1 proteins, the stability of TCF11-V5 and Nrf1a-V5 was assessed using cycloheximide chase assay in cells cotransfected with USP7-Flag or USP7-CS-Flag. In the absence of USP7-Flag, very little TCF11-V5 remained by 120 min after cycloheximide treatment (Fig. 2*F*). In contrast, the half-life of TCF11-V5 was prolonged by USP7-Flag, but not by the catalytically inactive form of USP7, suggesting that deubiquitinating activity of USP7 is required to stabilize Nrf1a (Fig. 2*F*). Similar results were obtained with cycloheximide chase of Nrf1a-V5 (Fig. 2*G*). Based on these results, we conclude that the stability of TCF11 and Nrf1a are extended by USP7.

### USP7 promotes deubiquitination of TCF11 and Nrf1a

**Figure 3:**
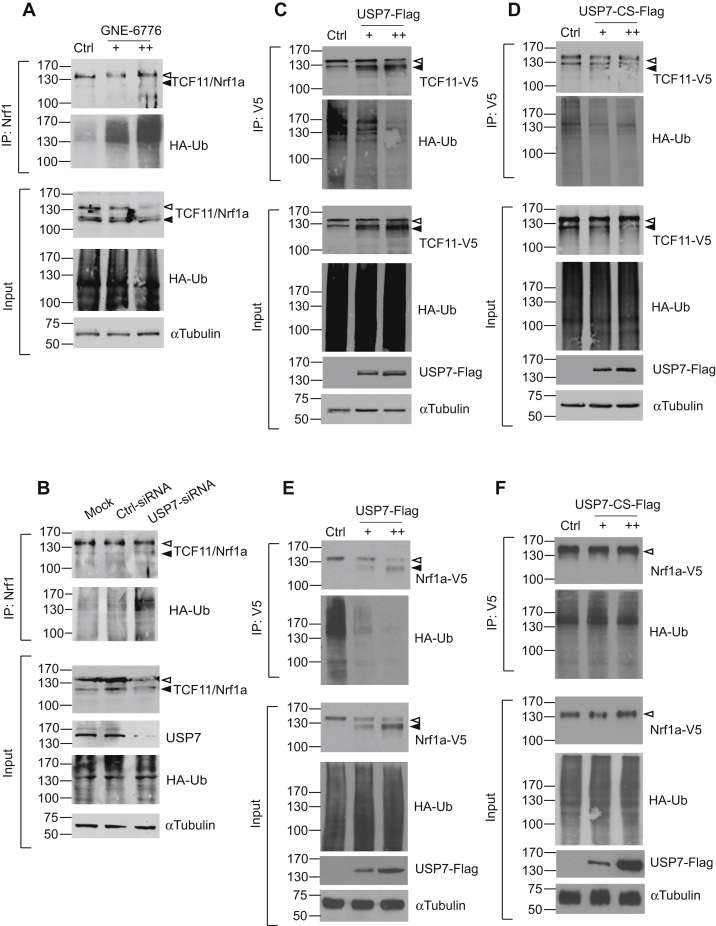
**USP7 promotes deubiquitination of Nrf1a and TCF11.***A*, HEK293 cells were transfected with 1 μg HA-Ub. After 24 h, cells were treated with GNE-6776 (0, 20, 40 μM) for 24 h, and endogenous TCF11/Nrf1a was immunoprecipitated with anti-TCF11 antibody followed by Western blotting with anti-HA-Ub antibody and anti-TCF11 antibody. Theunprocessed and processed forms of TCF11/Nrf1a are indicated by *open* or *filled arrowheads*, respectively. *Lower panels* show the input levels of endogenous TCF11/Nrf1a, HA-Ub, and alpha-Tubulin. *B*, HEK293 cells were transfected with 20 nM of a siRNA targeting USP7 or a negative control siRNA along with 1 μg HA-Ub. Cell lysates were then prepared for immunoprecipitation of endogenous TCF11/Nrf1a and immunoblotting for HA-Ub and TCF11/Nrf1a. *Lower panels* show the input levels of endogenous TCF11/Nrf1a, USP7, HA-Ub, and alpha-Tubulin. HEK293 cells were transfected with 1 μg HA-Ub and 1 μg TCF11-V5 (*C* and *D*) or Nrf1a-V5 (*E* and *F*) along with increasing amounts of (*C* and *E*) USP7-Flag (0, 1, 2 μg) or (*D* and *F*) USP7-CS-Flag (0, 1, 2 μg). Cell lysates were then prepared 24 h after and immunoprecipitated with anti-V5 mAb and analyzed for their ubiquitin content by immunoblotting with an anti-HA Ab. *Lower panels* show the input levels of TCF11-V5, Nrf1a-V5, HA-Ub, USP7-Flag, and USP7-CS-Flag.

Next, we examined the effects of USP7 on TCF11/Nrf1a ubiquitination. Endogenous TCF11/Nrf1a were immunoprecipitated from cells expressing HA-Ubiquitin and immunoblotted against HA-tag. Treatment with the USP7 inhibitor, GNE-6776, led to an increase in ubiquitination of endogenous TCF11/Nrf1a, indicating that USP7 has deubiquitination ability toward TCF11/Nrf1a (Fig. 3*A*). To confirm this finding, USP7 siRNA was transfected into HEK293 cells to knock down endogenous USP7 expression. Compared with control siRNA-transfected cells, the level of endogenous TCF11/Nrf1a ubiquitination was increased by knockdown of USP7 (Fig. 3*B*). To verify that USP7 deubiquitinates TCF11 and Nrf1a specifically, HEK293 cells were transfected with TCF11-V5 or Nrf1a-V5 and HA-tagged ubiquitin along with increasing amounts of Flag-tagged USP7, or USP7-CS, a dominant negative catalytically inactive form of USP7. V5-tagged proteins were immunoprecipitated from cell lysates with anti-V5 antibody followed by Western blotting with anti-HA antibody. Ubiquitinated forms of TCF11-V5 and Nrf1a-V5 were markedly reduced by overexpression of USP7 (Fig. 3, *C* and *E*). In contrast, levels of ubiquitinated TCF11-V5 and Nrf1a-V5 were not affected by expression of USP7-CS (Fig. 3, *D* and *F*). These results show that USP7 is able to stimulate deubiquitination of TCF11 and Nrf1a in cells and support the idea that USP7 regulates the stability of Nrf1 proteins through USP7 deubiquitinase activity.

### USP7 stabilizes Nrf1 in response to toxic metal exposure

**Figure 4:**
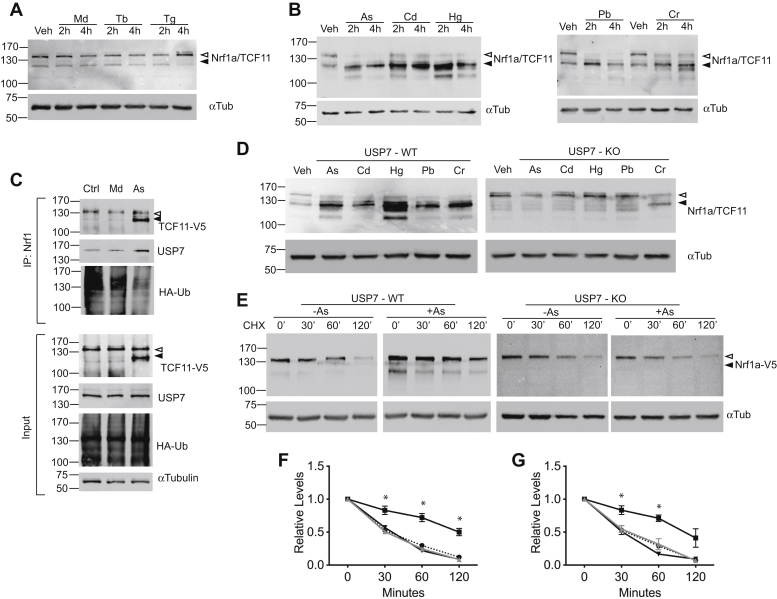
**USP7 stabilizes Nrf1a/TCF11 in response to toxic metal exposure.***A*, HEK293 cells were treated with vehicle (Veh), menadione (Md, 2.5 μM), thapsigargin (Tg, 1 μM), tertbutyl-hydroquinone (Tb, 10 μM) for 2 or 4 h. Cell lysates were then prepared and immunoblotted for endogenous TCF11/Nrf1a using anti-TCF11 antibody. Western blotting against alpha-tubulin was used for protein loading control. *B*, HEK293 cells were treated with vehicle (Veh), sodium arsenite (As, 25 μM), cadmium chloride (Cd, 100 μM), mercury chloride (Hg, 7.5 μM), lead acetate (Pb, 0.5 mM), or potassium dichromate (Cr, 10 μM) for 2 or 4 h. Cell lysates were immunoblotted for endogenous TCF11/Nrf1a using anti-TCF11 antibody, and alpha-tubulin was used for protein loading control. *C*, HEK293 cells transfected with 1 μg HA-Ub were treated with vehicle (Veh), menadione (Md, 2.5 μM), or sodium arsenite (As, 25 μM). Cell lysates were prepared 24 h after and immunoprecipitated with anti-TCF11 to pull down endogenous TCF11/Nrf1a and immunoblotted for endogenous USP7, HA-Ub. *Lower panels* show the input levels of TCF11/Nrf1a, USP7, HA-Ub, and alpha-tubulin. *D*, HCT116 USP7 wildtype cells or HCT116 USP7−/− cells were treated with sodium arsenite (As, 25 μM), cadmium chloride (Cd, 100 μM), mercury chloride (Hg, 5 μM), lead acetate (Pb, 0.5 mM), or potassium dichromate (Cr, 10 μM) for 2 h and immunoblotted for endogenous TCF11/Nrfa as described above. *E*, HCT116 USP7 wildtype cells or HCT116 USP7−/− cells were transfected with 1 μg Nrf1a-V5. After 24 h, cells were treated with vehicle (-As) or sodium arsenite (+As, 25 μM) along with cycloheximide (50 μg/ml) and were harvested at the indicated time points for Western blotting for the V5 tag using anti-V5 antibody, and alpha-tubulin was used as the loading control. *F*, graph shows quantitation of Nrf1a-V5 stability shown in *E*. Densitometric quantification of Nrf1a-V5 relative to alpha-tubulin at each time point; time 0 was set as 100%. Statistical analysis was performed using Student’s *t* test. Each point represents the mean ± SEM of remaining protein for three independent experiments, ∗*p* value <0.05. *G*, graph showing cycloheximide chase assay monitoring TCF11-V5 stability in HCT116 USP7 wildtype cells or HCT116 USP7−/− cells as described in *E*. Statistical analysis was performed using Student’s *t* test. Each point represents the mean ± SEM of remaining protein for three independent experiments, ∗*p* value <0.05.

Next, we sought to investigate whether USP7 plays a role in regulating Nrf1 levels in response to cellular stress. Arsenic has previously been shown to increase Nrf1 protein levels (39). Although arsenic has been associated with induction of oxidative stress in cells, treatment with menadione, tert-butylhydroquinone or thapsigargin, which are agents known to cause oxidative or ER stress did not lead to increased Nrf1 protein levels (Fig. 4*A*). Given that arsenic is a toxic metal, we investigated the effects other metal compounds on TCF11 and Nrf1a levels in HEK293 cells. Consistent with previous findings, HEK293 cells treated with arsenic showed upregulation of Nrf1 protein levels (Fig. 4*B*). In addition, endogenous Nrf1 levels were induced after a short exposure to cadmium and mercury, as well as lead and chromium suggesting that TCF11 and Nrf1a are stabilized by heavy metals (Fig. 4*B*, left and right panels). To examine the effects of arsenic on TCF11/Nrf1a ubiquitination, cells expressing HA-Ub were treated with menadione or arsenic, and endogenous TCF11/Nrf1a were immunoprecipitated for immunoblotting against HA-Ub and endogenous USP7. Compared with vehicle control and menadione, ubiquitination of endogenous TCF11/Nrf1a was decreased by arsenic (Fig. 4*C*). However, only a modest increase in USP7-TCF11/Nrf1a was detected by coimmunoprecipitation after arsenic treatment. To rule out possible cell-specific effects, expression of endogenous TCF11/Nrf1a in response to toxic metal exposure was examined in HCT116 cells, and similar results were obtained (Fig. 4*D*, left panel). To determine whether USP7 plays a role in this response, the effects of toxic metal exposure on endogenous TCF11/Nrf1a expression was examined in USP7 knockout HCT116 cells. Unlike wildtype cells, induction of TCF11/Nrf1a by toxic metal exposure was blunted or not observed in USP7 knockout cells (Fig. 4*D*, right panel). To confirm these observations, the half-life of V5-tagged Nrf1a in response to arsenic was evaluated by cycloheximide chase assays in USP7 wildtype and knockout cells. In USP7 wildtype cells, Nrf1a-V5 was stabilized by arsenic treatment (Fig. 4*E*, left panel and Fig. 4*F*). In contrast, stabilization by arsenic was blunted in USP7 knockout cells (Fig. 4*E*, right panel, and Fig. 4*F*). Similar results were obtained for V5-tagged TCF11 (Fig. 4*G*). Based on these results, we conclude that USP7 promotes stabilization of Nrf1 proteins in response to toxic metals.

### USP7 enhances transcriptional activation by Nrf1

**Figure 5:**
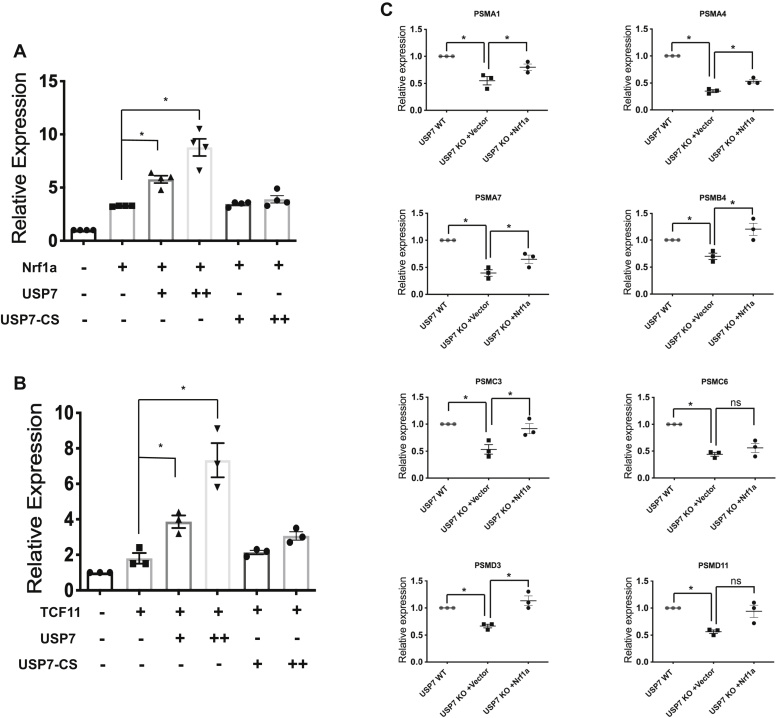
**USP7 enhances transcriptional activation by Nrf1a and TCF11.** HEK293 cells were cotransfected with 5 ng ARE-Luciferase, 0.1 ng TK-Renilla Luciferase control, along with 5 ng of (*A*) Nrf1a-V5 or (*B*) TCF11-V5 expression vector, 10 or 20 ng of USP7-Flag or USP7-CS-Flag expression vector. Firefly luciferase activity was normalized against Renilla Luciferase activity, and expression was calculated relative to normalized activity in the absence of Nrf1a and USP7 expression plasmid. Statistical analysis was performed using using Student’s *t* test, ∗*p* < 0.05. Values represent means ± SEM for three independent experiments each containing three replicates. *C*, expression of known Nrf1 target genes analyzed by RT-qPCR in USP7 wildtype, knockout, and knockout cells transfected with control or Nrf1a expression vector. The dot plots depict relative expression of indicated genes. *p* Values were calculated by Student’s *t* test (n = 3).

To investigate the significance of the interaction between USP7 and Nrf1, we used a transient reporter assay to examine whether USP7 affects transcriptional activity of TCF11 and Nrf1a. HEK293 cells were transfected with Nrf1a-V5 and a Nrf1-responsive luciferase reporter plasmid along with USP7, or USP7-CS expression plasmids. Luciferase reporter expression was enhanced by coexpression of USP7 in a dose-dependent manner (Fig. 5*A*). In contrast, coexpression of the catalytically inactive USP7-CS had no effect on reporter activation by Nrf1a (Fig. 5*A*). Similarly, coexpression of USP7, but not USP7-CS, enhanced reporter expression by TCF11-V5 (Fig. 5*B*). To further confirm the role of USP7 on Nrf1-mediated transcriptional activation, we examined the expression of known Nrf1-target genes in USP7 knockout cells. Compared with wildtype cells, expression of various proteasome genes that were examined was markedly reduced in USP7 knockout cells (Fig. 5*C*), and transfection of Nrf1a cDNA fully or partially restored their expression in knockout cells. These results indicate that the transacting function of TCF11 and Nrf1a is enhanced through increased protein levels enabled by USP7.

### Inhibition of USP7 impairs Nrf1a-mediated protection against arsenic-induced cytotoxicity

**Figure 6:**
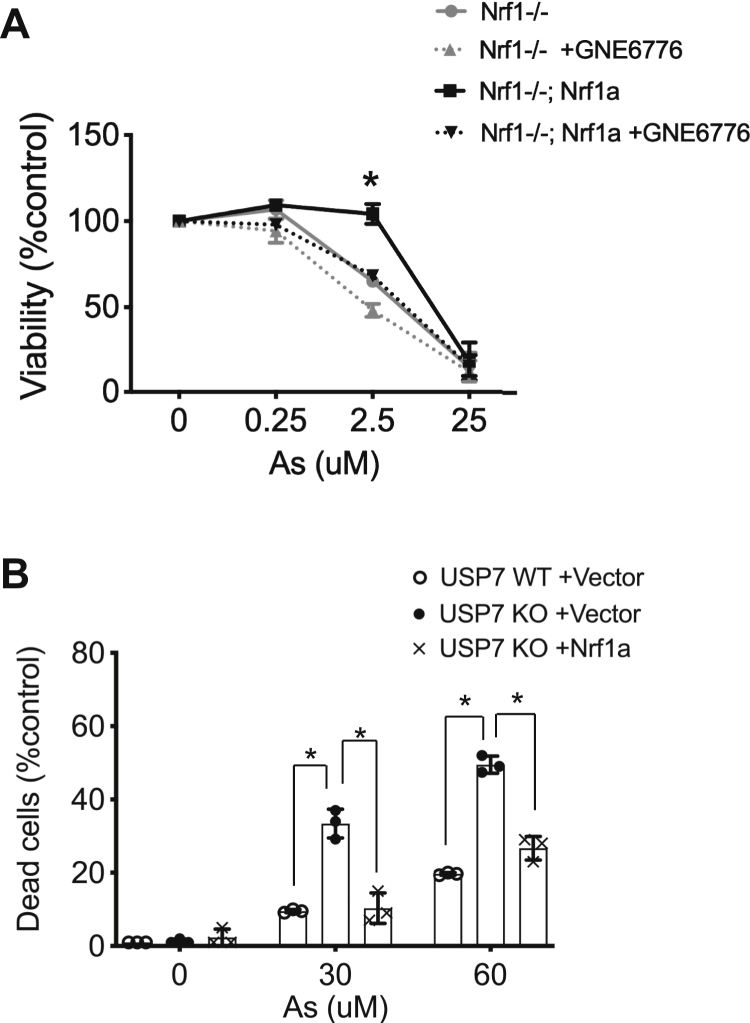
**Inhibition of USP7 impairs Nrf1a-mediated protection against arsenic-induced cytotoxicity.***A*, Nrf1−/− MEF cells or Nrf1−/− expressing Nrf1a were cultured with 0, 0.25, 2.5, or 25 μM sodium arsenite along with vehicle or 20 μM GNE-6776. After 24 h, WST-1 was added, and absorbance for formation of formazan product was measured at 450 nm (with reference absorbance at 620 nm and corrections using blanks). Percent viability was calculated as (sample absorbance/control absorbance) × 100%. Data represent means ± SEM for three independent experiments each containing three replicates. Statistical analysis between cell lines was carried out using one-way ANOVA. (∗) represents *p* < 0.05. *B*, USP7 wildtype or knockout HCT116 cells transfected with control or Nrf1a expression vector were cultured with 0, 30, or 60 μM sodium arsenite. After 24 h, trypan blue exclusion assays were done. Percent dead cells was calculated as (dead cells/dead cells + live cells) × 100%. Data represent means ± SEM for three independent experiments each containing three replicates. Statistical analysis was done using Student’s *t* test. (∗) represents *p* < 0.05.

To further determine the functional significance of Nrf1 as a USP7 substrate, we sought to determine the influence of USP7 on Nrf1-mediated cell survival in response to toxic metal exposure. For this purpose, we measured the effects of blocking USP7 function on the viability of wildtype and Nrf1 knockout cells in response to arsenic treatment for 24 h. We used a pair of Nrf1 knockout mouse embryonic fibroblast cells, one of which is reconstituted with wildtype Nrf1a cDNA. As expected, Nrf1 knockout cells were more sensitive to arsenic-induced toxicity compared with knockout cells expressing Nrf1a (Fig. 6*A*). Next, we investigated the effects of USP7 inhibition. Compared with Nrf1 knockout cells, knockout cells expressing Nrf1a treated with vehicle-control were more resistant to the cytotoxic effects of arsenic. However, pretreatment with GNE-6776 rendered Nrf1a-expressing knockout cells sensitive to arsenic toxicity (Fig. 6*A*). To substantiate these findings, we also examined viability of USP7 knockout cells in response to arsenic. Cells treated with increasing concentrations of arsenic were monitored after 24 h. USP7 knockout cells were more sensitive to arsenic than wildtype cells (Fig. 6*B*). To establish a role for Nrf1 in arsenic sensitivity, USP7 knockout cells were transfected with Nrf1a-V5, or empty vector as experimental control. In comparison with control transfected cells, Nrf1a-transfected cells exhibited significantly lower cytotoxicity to arsenic. These findings indicate that loss of USP7 function reduces Nrf1-mediated resistance to toxicity induced by arsenic.

## Discussion

USP7 is an abundant deubiquitinating enzyme originally identified as a binding partner of ICP0, a transactivator protein found in herpes virus (32). USP7 has subsequently been shown to be involved in multiple cellular processes such as DNA damage and repair, epigenetic regulation, and immune responses. In addition to its well-characterized role in regulating tumor protein p53 and MDM2 levels, USP7 has a broad range of substrates, and it has also shown to modulate the stability of various other proteins such as Phosphatase and tensin homolog, Forkhead box 4, and Claspin (40, 41, 42). Here, we identify USP7 as a novel regulator of Nrf1 protein stability. USP7 binds TCF11 and Nrf1a and controls their stability by direct deubiquitination. These effects of USP7 on TCF11 and Nrf1a were confirmed by expression of a dominant-negative catalytically inactive mutant USP7 and by RNAi-mediated knockdown and pharmacologic inhibition of endogenous USP7 function. Overexpression of wildtype, but not mutant, USP7 enhanced transactivation by TCF11 and Nrf1a in a luciferase reporter assay. Together, these results support the idea that USP7 can modulate Nrf1 function in cells.

Our results show that TCF11 and Nrf1a proteins are stabilized by USP7 in response to arsenic and other heavy metals. Although it was previously demonstrated that arsenic leads to higher stability and transcriptional activity of Nrf1, the mechanism underlying Nrf1-mediated responses to arsenic exposure was not shown (39). Evidence indicates that multifactorial mechanism is involved in heavy metal–induced cellular stress, and one of them involves generation of reactive oxygen species through metal-induced reduction–oxidation, reaction which leads to oxidative damage of cellular components (43). However, the observation that TCF11 and Nrf1a levels were not induced by reactive oxygen species–generating agents, TBHQ and menadione, suggests that USP7-mediated stabilization of Nrf1 by toxic metals is triggered by a different mechanism. In addition to oxidative stress, various heavy metals have electron-sharing affinity that results in its covalent attachment to thiol residues of cellular proteins (44). Such property disrupts structural and functional integrity of substrate proteins that could lead to accumulation of misfolded and nonfunctioning proteins (45, 46, 47, 48). Cadmium, lead, and mercury, for example, have been shown to form complexes with thiol functional groups on proteins and inhibit refolding of denatured protein, whereas arsenic and cadmium are also known to interfere with chaperone-mediated refolding of proteins (49, 50). It is also thought that arsenic and cadmium impede folding of nascent polypeptides causing accumulation of misfolded proteins (51, 52, 53, 54). Thus, it is possible that USP7 may play a role in mitigating other types of cellular stress through Nrf1 in response to toxic metal exposure. Currently, however, the full spectrum of genes induced through the USP7-Nrf1 pathway is not known and requires further experimentation.

While the current study cannot rule out direct effects of toxic metals on Nrf1-USP7 interaction, USP7 expression was not upregulated by arsenic (data not shown). Thus, the mechanism by which exposure to arsenic and other metals trigger USP7 regulation of Nrf1 is not known. One possibility is that USP7 associates with Nrf1 proteins under normal conditions, and its deubiquitinating enzyme activity is turned on by posttranslational modification in response to stimuli induced by toxic metal compounds. Alternatively, Nrf1 proteins themselves undergo posttranslational modification as a result of toxic metal exposure, which then signals the recruitment of USP7 binding and subsequent deubiquitination. In this regard, it has been reported that heavy metal exposure leads to rapid increase of O-GlcNAcylation of numerous intracellular proteins, and O-GlcNAc (O-linked β-N-acetylglucosamine) modifications are found to serve as recruiting signals for deubiquitinase proteins such as BRCA1 associated protein-1 (55, 56). It is interesting to note that Nrf1a undergoes O-GlcNAcylation by O-GlcNAc transferase in response to cellular stresses and the addition of O-GlcNAc results in decreased ubiquitination and degradation of Nrf1a (37). It remains to be shown whether O-GlcNAc-mediated stabilization of TCF11 or Nrf1a involves USP7. In addition to USP7, another USP-type deubiquitinase, USP15, was also shown to stabilize Nrf1 (57). Thus, two different deubiquitinase enzymes contribute to enhancing Nrf1a function. It would be of interest to determine whether USP15-mediated stabilization of Nrf1 is induced similarly as USP7, as well as determine whether they represent discrete pathways that antagonizes the actions of ubiquitin ligases such as F-box/WD repeat-containing protein 7 and beta-transducin repeats-containing proteins that have been shown to promote destabilization of Nrf1a (25, 26).

In summary, our findings identify USP7 as a positive regulator of Nrf1 expression. This suggests a model where ubiquitination and deubiquitination, regulated by E3 ligases and deubiquitinating enzymes, controls the Nrf1 pathway and suggests USP7 as a potential target to modulate Nrf1 function. Although USP7 is recognized for its role in controlling various aspects of cellular processes such cell survival, cell cycle, and viral infection, our results here provide a new context in which USP7 may play a role in signaling through Nrf1 in response to toxic metals and metalloids common to the environment that are associated with various health problems in humans.

## Experimental procedures

### Reagents

Dulbecco’s modified Eagle’s medium (DMEM), streptomycin, penicillin, and fetal bovine serum (FBS) were purchased from Invitrogen (Carlsbad). Bradford protein assay reagent was from Bio-Rad (Hercules). BioT was purchased from Bioland Scientific. Dual Reporter Assay Kit was from Promega. ImmunoPure streptavidin and enhanced chemiluminescence substrate kit were from Pierce Biotechnology. WST-1 reagent, sodium arsenite, cadmium chloride, thapsigargin, mercury chloride, and lead acetate were purchased from Sigma-Aldrich. GNE-6776 (USP7 inhibitor) was from Aobious Inc, and USP7-siRNA-27mer was purchased from Origene. All other general chemicals for buffers and culture media were purchased from Thermo Fisher Scientific and/or Sigma-Aldrich. Primary antibodies against Tubulin-A (3873, mouse mAb), USP7 (3277, Rabbit pAb), Flag-Tag (14793, Rabbit mAb), and HA-Tag (2367, mAb) were purchased from Cell Signaling Technology. Horseradish peroxidase–linked anti-rabbit IgG (7074) and anti-mouse IgG (7076) antibodies were also from Cell Signaling Technology. Mouse monoclonal anti-V5 tag (MA5-15253) was from Thermo Fisher. Antibodies from Cell Signaling Technology and Thermo Fisher have been tested and validated on the lot level by manufacturer’s in-house scientists for specificity, sensitivity, and reproducibility. Antibody against TCF11 and Nrf1a is from Cell Signaling (D5B10), and it has been validated for specificity using Nrf1 knockout cells or mouse tissues (19). Human USP7 (−/−) knockout HCT116 cells were obtained from Horizon Discovery.

### Plasmids

ARE-Luciferase is from Promega. Flag-tagged and HA-tagged USP7 and USP7-CS plasmids were obtained from Addgene. The catalytically inactive mutant of USP7 was generated by Maertens *et al.* (58). V5-tagged Nrf1 and Ub-HA expression plasmids were described (37).

### Western blotting

Cells were lysed in cold RIPA buffer (50 mM Tris-HCl pH 7.4, 150 mM NaCl, 1% Triton X-100, 1% sodium deoxycholate, 0.1% SDS, 1X Protease Inhibitor). Lysates were cleared by centrifugation for 15 min at 4 °C, and protein concentrations were determined by Bradford assay. An equal volume of 2× SDS sample buffer (100 mM Tris, pH 6.8, 25% glycerol, 2% SDS, 0.01% bromophenol blue, 10% 2-mercaptoethanol) was added to cell lysates, and the mixture was boiled for 5 min. Proteins were electrophoresed on SDS-PAGE gels and transferred onto nitrocellulose membranes. Membranes were then blocked in 5% nonfat dry milk in TBS-T (150 mM NaCl, 50 mM Tris-HCl pH 8.0, and 0.05% Tween 20) at room temperature for 1 h and then incubated with the indicated primary antibodies at 1:1000 dilution (unless otherwise indicated) overnight at 4 °C followed by incubation with 1:2000 dilution of horseradish peroxidase–conjugated secondary anti-rabbit or anti-mouse antibody. Antibody–antigen complexes on the blots were detected using chemiluminescent detection system. Densitometric analysis was performed using the Un-Scan-It Gel Analysis software for normalization.

### Transfection and luciferase assays

DNA transfections were done using BioT reagent from Bioland Scientific for HEK293 cells and Lipofectamine 3000 from Thermo Fisher Scientific for HCT116 cells according to the manufacturer’s protocol. For luciferase assays, HEK293T cells were seeded onto a 24-well plate 24 h before transfections. Cellular extracts were prepared 24 h after transfection, and Firefly- and Renilla-luciferase activities were measured using Dual Reporter Assay Kit.

### Coimmunoprecipitation

Subconfluent HEK293T or HCT116 cells were transfected with the indicated amount of expression vectors using BioT according to manufacturer’s protocol. Cells were lysed in buffer containing 2% SDS, 150 mM NaCl, 10 mM Tris-HCl (pH 8.0), and 1 mM DTT. Lysates prepared in SDS-containing buffer were diluted 5-fold with buffer lacking SDS. Diluted lysates were subjected to preclearing with protein-G Sepharose beads by incubation in the cold for 1 h. The protein samples were incubated with 2 to 5 μg of primary antibodies or IgG as a control overnight at 4 °C. After overnight incubation, protein-G Sepharose beads were added and incubated at 4 °C for 1 h. Beads were then collected by brief centrifugation and washed extensively with RIPA buffer. Proteins were eluted in 1× SDS sample buffer and heated at 95 °C for 5 min. The samples were separated by SDS-PAGE and transferred onto a nitrocellulose membrane, followed by immunoblotting with indicated primary antibodies and horseradish peroxidase–conjugated secondary antibodies. Detection of peroxidase signal was performed using the chemiluminescence method.

### Cycloheximide chase assays

HEK293T or HCT116 cells expressing various constructs indicated in the figures were incubated with 50 μg/ml cycloheximide in DMEM at 37 °C. At the times indicated, cells were harvested and lysates were prepared for immunoblotting.

### RNA isolation and RT-PCR

Total RNA was extracted with Zymo DirectZol RNA Miniprep (Zymo Research), and cDNA was synthesized using iScript Advanced cDNA Synthesis Kit (Bio-Rad). Quantitative RT-PCR was performed using Roche FastStart DNA Green Master Mix (Roche LifeScience) with previously described primers (37) or KiCqStart predesigned primers (Sigma-Aldrich) in a QuantStudio 3 Real-Time PCR system running QuantStudio Design and Analysis Software (v1.5.1, Applied Biosystems). PCR cycling conditions consist of 95 °C for 10 min and 40 cycles of 95 °C for 10 s and 60 °C for 30 s. Results were calculated using the ΔΔCT method with ALAS1 as reference gene.

### Cell viability assay

Mouse embryonic fibroblast cells were seeded at a density of 10^4^ cells/well/100 μl media in 96-well plates. After overnight growth, cells were incubated with sodium arsenite at different concentrations for 24 h at 37 °C. At the end of incubation, the water-soluble tetrazolium salts (WST-1) conversion assay was done by adding 10 μl of WST-1 reagent. After incubation for 1 h at 37 °C, absorbance reading at 450 nm (with correction using readings at 620 nm) was measured using a multiwell plate reader (Molecular Devices Corp). Wells containing media alone were used as blanks to subtract absorption by media components. Triplicate values were averaged, and SEM was calculated and graphed with Prism software (GraphPad). Cell viability was determined as follows: Viability (%) = (Absorbance of sample/Absorbance of control) × 100%. HCT116 cells were seeded at a density of 0.75 × 10^6^ cells/well in six-well plates and incubated with the indicated concentrations of arsenic. Cells were harvested after 24 h of incubation. Trypan blue solution was added to the cell suspensions in a 1:1 ratio, and the percentage of living cells and dead cells was analyzed on a DeNovix automated cell counter.

### Statistical analysis

Data are expressed as means ± SEM. Statistical analyses using Student’s *t* test or one-way ANOVA were done with Prism software (GraphPad). ∗ indicates *p* values <0.05 and considered significant.

## Data availability

All data are contained within the figures.

## Conflict of interest

The authors declare that they have no conflicts of interest with the contents of this article.

## References

[1] Chan J.Y., Kwong M., Lu R., Chang J., Wang B., Yen T.S., Kan Y.W. (1998). Targeted disruption of the ubiquitous CNC-bZIP transcription factor, Nrf-1, results in anemia and embryonic lethality in mice. EMBO J..

[2] Kobayashi A., Tsukide T., Miyasaka T., Morita T., Mizoroki T., Saito Y., Ihara Y., Takashima A., Noguchi N., Fukamizu A., Hirotsu Y., Ohtsuji M., Katsuoka F., Yamamoto M. (2011). Central nervous system-specific deletion of transcription factor Nrf1 causes progressive motor neuronal dysfunction. Genes Cells.

[3] Lee C.S., Lee C., Hu T., Nguyen J.M., Zhang J., Martin M.V., Vawter M.P., Huang E.J., Chan J.Y. (2011). Loss of nuclear factor E2-related factor 1 in the brain leads to dysregulation of proteasome gene expression and neurodegeneration. Proc. Natl. Acad. Sci. U. S. A..

[4] Xu Z., Chen L., Leung L., Yen T.S., Lee C., Chan J.Y. (2005). Liver-specific inactivation of the Nrf1 gene in adult mouse leads to nonalcoholic steatohepatitis and hepatic neoplasia. Proc. Natl. Acad. Sci. U. S. A..

[5] Kim H.M., Han J.W., Chan J.Y. (2016). Nuclear factor erythroid-2 like 1 (NFE2L1): Structure, function and regulation. Gene.

[6] Kwong M., Kan Y.W., Chan J.Y. (1999). The CNC basic leucine zipper factor, Nrf1, is essential for cell survival in response to oxidative stress-inducing agents. Role for Nrf1 in gamma-gcs(l) and gss expression in mouse fibroblasts. J. Biol. Chem..

[7] Lee C.S., Ho D.V., Chan J.Y. (2013). Nuclear factor-erythroid 2-related factor 1 regulates expression of proteasome genes in hepatocytes and protects against endoplasmic reticulum stress and steatosis in mice. FEBS J..

[8] Myhrstad M.C., Husberg C., Murphy P., Nordstrom O., Blomhoff R., Moskaug J.O., Kolsto A.B. (2001). TCF11/Nrf1 overexpression increases the intracellular glutathione level and can transactivate the gamma-glutamylcysteine synthetase (GCS) heavy subunit promoter. Biochim. Biophys. Acta.

[9] Ohtsuji M., Katsuoka F., Kobayashi A., Aburatani H., Hayes J.D., Yamamoto M. (2008). Nrf1 and Nrf2 play distinct roles in activation of antioxidant response element-dependent genes. J. Biol. Chem..

[10] Radhakrishnan S.K., Lee C.S., Young P., Beskow A., Chan J.Y., Deshaies R.J. (2010). Transcription factor Nrf1 mediates the proteasome recovery pathway after proteasome inhibition in mammalian cells. Mol. Cell.

[11] Kim J., Xing W., Wergedal J., Chan J.Y., Mohan S. (2010). Targeted disruption of nuclear factor erythroid-derived 2-like 1 in osteoblasts reduces bone size and bone formation in mice. Physiol. Genomics.

[12] Narayanan K., Ramachandran A., Peterson M.C., Hao J., Kolsto A.B., Friedman A.D., George A. (2004). The CCAAT enhancer-binding protein (C/EBP)beta and Nrf1 interact to regulate dentin sialophosphoprotein (DSPP) gene expression during odontoblast differentiation. J. Biol. Chem..

[13] Oh D.H., Rigas D., Cho A., Chan J.Y. (2012). Deficiency in the nuclear-related factor erythroid 2 transcription factor (Nrf1) leads to genetic instability. FEBS J..

[14] Xing W., Singgih A., Kapoor A., Alarcon C.M., Baylink D.J., Mohan S. (2007). Nuclear factor-E2-related factor-1 mediates ascorbic acid induction of osterix expression via interaction with antioxidant-responsive element in bone cells. J. Biol. Chem..

[15] Luna L., Skammelsrud N., Johnsen O., Abel K.J., Weber B.L., Prydz H., Kolsto A.B. (1995). Structural organization and mapping of the human TCF11 gene. Genomics.

[16] Koizumi S., Irie T., Hirayama S., Sakurai Y., Yashiroda H., Naguro I., Ichijo H., Hamazaki J., Murata S. (2016). The aspartyl protease DDI2 activates Nrf1 to compensate for proteasome dysfunction. Elife.

[17] Radhakrishnan S.K., den Besten W., Deshaies R.J. (2014). p97-Dependent retrotranslocation and proteolytic processing govern formation of active Nrf1 upon proteasome inhibition. Elife.

[18] Steffen J., Seeger M., Koch A., Kruger E. (2010). Proteasomal degradation is transcriptionally controlled by TCF11 via an ERAD-dependent feedback loop. Mol. Cell.

[19] Wang W., Chan J.Y. (2006). Nrf1 is targeted to the endoplasmic reticulum membrane by an N-terminal transmembrane domain. Inhibition of nuclear translocation and transacting function. J. Biol. Chem..

[20] Zhang Y., Crouch D.H., Yamamoto M., Hayes J.D. (2006). Negative regulation of the Nrf1 transcription factor by its N-terminal domain is independent of Keap1: Nrf1, but not Nrf2, is targeted to the endoplasmic reticulum. Biochem. J..

[21] Caterina J.J., Donze D., Sun C.W., Ciavatta D.J., Townes T.M. (1994). Cloning and functional characterization of LCR-F1: A bZIP transcription factor that activates erythroid-specific, human globin gene expression. Nucleic Acids Res..

[22] Husberg C., Murphy P., Martin E., Kolsto A.B. (2001). Two domains of the human bZIP transcription factor TCF11 are necessary for transactivation. J. Biol. Chem..

[23] Kwong E.K., Kim K.M., Penalosa P.J., Chan J.Y. (2012). Characterization of Nrf1b, a novel isoform of the nuclear factor-erythroid-2 related transcription factor-1 that activates antioxidant response element-regulated genes. PLoS One.

[24] Wang W., Kwok A.M., Chan J.Y. (2007). The p65 isoform of Nrf1 is a dominant negative inhibitor of ARE-mediated transcription. J. Biol. Chem..

[25] Biswas M., Phan D., Watanabe M., Chan J.Y. (2011). The Fbw7 tumor suppressor regulates nuclear factor E2-related factor 1 transcription factor turnover through proteasome-mediated proteolysis. J. Biol. Chem..

[26] Tsuchiya Y., Morita T., Kim M., Iemura S., Natsume T., Yamamoto M., Kobayashi A. (2011). Dual regulation of the transcriptional activity of Nrf1 by beta-TrCP- and Hrd1-dependent degradation mechanisms. Mol. Cell. Biol..

[27] Sowa M.E., Bennett E.J., Gygi S.P., Harper J.W. (2009). Defining the human deubiquitinating enzyme interaction landscape. Cell.

[28] Everett R.D., Meredith M., Orr A., Cross A., Kathoria M., Parkinson J. (1997). A novel ubiquitin-specific protease is dynamically associated with the PML nuclear domain and binds to a herpesvirus regulatory protein. EMBO J..

[29] Nijman S.M., Luna-Vargas M.P., Velds A., Brummelkamp T.R., Dirac A.M., Sixma T.K., Bernards R. (2005). A genomic and functional inventory of deubiquitinating enzymes. Cell.

[30] Rawat R., Starczynowski D.T., Ntziachristos P. (2019). Nuclear deubiquitination in the spotlight: The multifaceted nature of USP7 biology in disease. Curr. Opin. Cell Biol..

[31] Cummins J.M., Rago C., Kohli M., Kinzler K.W., Lengauer C., Vogelstein B. (2004). Tumour suppression: Disruption of HAUSP gene stabilizes p53. Nature.

[32] Boutell C., Canning M., Orr A., Everett R.D. (2005). Reciprocal activities between herpes simplex virus type 1 regulatory protein ICP0, a ubiquitin E3 ligase, and ubiquitin-specific protease USP7. J. Virol..

[33] Colleran A., Collins P.E., O'Carroll C., Ahmed A., Mao X., McManus B., Kiely P.A., Burstein E., Carmody R.J. (2013). Deubiquitination of NF-kappaB by ubiquitin-specific protease-7 promotes transcription. Proc. Natl. Acad. Sci. U. S. A..

[34] Cummins J.M., Vogelstein B. (2004). HAUSP is required for p53 destabilization. Cell Cycle.

[35] Hao Y.H., Fountain M.D., Fon Tacer K., Xia F., Bi W., Kang S.H., Patel A., Rosenfeld J.A., Le Caignec C., Isidor B., Krantz I.D., Noon S.E., Pfotenhauer J.P., Morgan T.M., Moran R. (2015). USP7 acts as a molecular rheostat to promote WASH-dependent endosomal protein recycling and is mutated in a human neurodevelopmental disorder. Mol. Cell.

[36] Lecona E., Rodriguez-Acebes S., Specks J., Lopez-Contreras A.J., Ruppen I., Murga M., Munoz J., Mendez J., Fernandez-Capetillo O. (2016). USP7 is a SUMO deubiquitinase essential for DNA replication. Nat. Struct. Mol. Biol..

[37] Han J.W., Valdez J.L., Ho D.V., Lee C.S., Kim H.M., Wang X., Huang L., Chan J.Y. (2017). Nuclear factor-erythroid-2 related transcription factor-1 (Nrf1) is regulated by O-GlcNAc transferase. Free Radic. Biol. Med..

[38] Turnbull A.P., Ioannidis S., Krajewski W.W., Pinto-Fernandez A., Heride C., Martin A.C.L., Tonkin L.M., Townsend E.C., Buker S.M., Lancia D.R., Caravella J.A., Toms A.V., Charlton T.M., Lahdenranta J., Wilker E. (2017). Molecular basis of USP7 inhibition by selective small-molecule inhibitors. Nature.

[39] Zhao R., Hou Y., Xue P., Woods C.G., Fu J., Feng B., Guan D., Sun G., Chan J.Y., Waalkes M.P., Andersen M.E., Pi J. (2011). Long isoforms of NRF1 contribute to arsenic-induced antioxidant response in human keratinocytes. Environ. Health Perspect..

[40] Faustrup H., Bekker-Jensen S., Bartek J., Lukas J., Mailand N. (2009). USP7 counteracts SCFbetaTrCP- but not APCCdh1-mediated proteolysis of Claspin. J. Cell Biol..

[41] Song M.S., Salmena L., Carracedo A., Egia A., Lo-Coco F., Teruya-Feldstein J., Pandolfi P.P. (2008). The deubiquitinylation and localization of PTEN are regulated by a HAUSP-PML network. Nature.

[42] van der Horst A., de Vries-Smits A.M., Brenkman A.B., van Triest M.H., van den Broek N., Colland F., Maurice M.M., Burgering B.M. (2006). FOXO4 transcriptional activity is regulated by monoubiquitination and USP7/HAUSP. Nat. Cell Biol..

[43] Liochev S.I. (1999). The mechanism of “Fenton-like” reactions and their importance for biological systems. A biologist's view. Met. Ions Biol. Syst..

[44] Stohs S.J., Bagchi D. (1995). Oxidative mechanisms in the toxicity of metal ions. Free Radic. Biol. Med..

[45] Hiramatsu N., Kasai A., Du S., Takeda M., Hayakawa K., Okamura M., Yao J., Kitamura M. (2007). Rapid, transient induction of ER stress in the liver and kidney after acute exposure to heavy metal: Evidence from transgenic sensor mice. FEBS Lett..

[46] Letelier M.E., Lepe A.M., Faundez M., Salazar J., Marin R., Aracena P., Speisky H. (2005). Possible mechanisms underlying copper-induced damage in biological membranes leading to cellular toxicity. Chem. Biol. Interact..

[47] Shinkai Y., Yamamoto C., Kaji T. (2010). Lead induces the expression of endoplasmic reticulum chaperones GRP78 and GRP94 in vascular endothelial cells via the JNK-AP-1 pathway. Toxicol. Sci..

[48] Valko M., Morris H., Cronin M.T. (2005). Metals, toxicity and oxidative stress. Curr. Med. Chem..

[49] Jacobson T., Navarrete C., Sharma S.K., Sideri T.C., Ibstedt S., Priya S., Grant C.M., Christen P., Goloubinoff P., Tamas M.J. (2012). Arsenite interferes with protein folding and triggers formation of protein aggregates in yeast. J. Cell Sci..

[50] Jacobson T., Priya S., Sharma S.K., Andersson S., Jakobsson S., Tanghe R., Ashouri A., Rauch S., Goloubinoff P., Christen P., Tamas M.J. (2017). Cadmium causes misfolding and aggregation of cytosolic proteins in yeast. Mol. Cell. Biol..

[51] Jin Y.H., Dunlap P.E., McBride S.J., Al-Refai H., Bushel P.R., Freedman J.H. (2008). Global transcriptome and deletome profiles of yeast exposed to transition metals. PLoS Genet..

[52] Pan X., Reissman S., Douglas N.R., Huang Z., Yuan D.S., Wang X., McCaffery J.M., Frydman J., Boeke J.D. (2010). Trivalent arsenic inhibits the functions of chaperonin complex. Genetics.

[53] Sharma S.K., Goloubinoff P., Christen P. (2008). Heavy metal ions are potent inhibitors of protein folding. Biochem. Biophys. Res. Commun..

[54] Thorsen M., Lagniel G., Kristiansson E., Junot C., Nerman O., Labarre J., Tamas M.J. (2007). Quantitative transcriptome, proteome, and sulfur metabolite profiling of the *Saccharomyces cerevisiae* response to arsenite. Physiol. Genomics.

[55] Ruan H.B., Han X., Li M.D., Singh J.P., Qian K., Azarhoush S., Zhao L., Bennett A.M., Samuel V.T., Wu J., Yates J.R., Yang X. (2012). O-GlcNAc transferase/host cell factor C1 complex regulates gluconeogenesis by modulating PGC-1alpha stability. Cell Metab..

[56] Zachara N.E., O'Donnell N., Cheung W.D., Mercer J.J., Marth J.D., Hart G.W. (2004). Dynamic O-GlcNAc modification of nucleocytoplasmic proteins in response to stress. A survival response of mammalian cells. J. Biol. Chem..

[57] Fukagai K., Waku T., Chowdhury A., Kubo K., Matsumoto M., Kato H., Natsume T., Tsuruta F., Chiba T., Taniguchi H., Kobayashi A. (2016). USP15 stabilizes the transcription factor Nrf1 in the nucleus, promoting the proteasome gene expression. Biochem. Biophys. Res. Commun..

[58] Maertens G.N., El Messaoudi-Aubert S., Elderkin S., Hiom K., Peters G. (2010). Ubiquitin-specific proteases 7 and 11 modulate polycomb regulation of the INK4a tumour suppressor. EMBO J..

